# Dry weather induces outbreaks of human West Nile virus infections

**DOI:** 10.1186/1471-2334-10-38

**Published:** 2010-02-24

**Authors:** Guiming Wang, Richard B Minnis, Jerrold L Belant, Charles L Wax

**Affiliations:** 1 Department of Wildlife, Fisheries and Aquaculture, Mississippi State University, Mail stop 9690, Mississippi State, Mississippi 39762, USA; 2 Department of Geosciences, Mississippi State University, Mail stop 5448, Mississippi State, Mississippi 39762, USA; 3 United States Department of Agriculture Animal and Plant Health Inspection Service Wildlife Services, 4700 River Road, Riverdale MD 20737, USA

## Abstract

**Background:**

Since its first occurrence in the New York City area during 1999, West Nile virus (WNV) has spread rapidly across North America and has become a major public health concern in North America. By 2002, WNV was reported in 40 states and the District of Columbia with 4,156 human and 14,539 equine cases of infection. Mississippi had the highest human incidence rate of WNV during the 2002 epidemic in the United States. Epidemics of WNV can impose enormous impacts on local economies. Therefore, it is advantageous to predict human WNV risks for cost-effective controls of the disease and optimal allocations of limited resources. Understanding relationships between precipitation and WNV transmission is crucial for predicting the risk of the human WNV disease outbreaks under predicted global climate change scenarios.

**Methods:**

We analyzed data on the human WNV incidences in the 82 counties of Mississippi in 2002, using standard morbidity ratio (SMR) and Bayesian hierarchical models, to determine relationships between precipitation and human WNV risks. We also entertained spatial autocorrelations of human WNV risks with conditional autocorrelative (CAR) models, implemented in WinBUGS 1.4.3.

**Results:**

We observed an inverse relationship between county-level human WNV incidence risk and total annual rainfall during the previous year. Parameters representing spatial heterogeneity in the risk of human exposure to WNV improved model fit. Annual precipitation of the previous year was a predictor of spatial variation of WNV risk.

**Conclusions:**

Our results have broad implications for risk assessment of WNV and forecasting WNV outbreaks. Assessing risk of vector-born infectious diseases will require understanding of complex ecological relationships. Based on the climatologically characteristic drought occurrence in the past and on climate model predictions for climate change and potentially greater drought occurrence in the future, we suggest that the frequency and relative risk of WNV outbreaks could increase.

## Background

Initially detected in Uganda in 1937, West Nile virus (WNV) spread across Africa to the Middle East, West Asia, and eastern Europe [1,2]. The first occurrence of WNV in the Western Hemisphere was in the New York City area during 1999, where 59 patients were hospitalized with WNV infection during August and September [2]. West Nile virus spread rapidly across North America and by 2002, was reported in 40 states and the District of Columbia of the United States (US) with 4,156 human and 14,539 equine cases of infection [3]. West Nile virus infection can cause neuroinvasive diseases (e.g., encephalitis) and even human fatalities [3]. Additionally, an epidemic of WNV can impose enormous impacts on local economies. For instance, the estimated short-term economic cost incurred from the 2002 WNV epidemic in Louisiana was $20.1 million [4]. West Nile virus disease will continue to be a public health concern in the foreseeable future; therefore, the assessment and prediction of human WNV risk within an administrative unit (e.g., county) is critical for effective WNV control and prevention and resource allocation [5].

Understanding ecological factors influencing the transmission of WNV can help predict human WNV risk and improve effectiveness of control measures [1]. Birds are the predominant hosts of WNV, whereas mammals (including humans and equines) are accidental, dead-end hosts [6]. West Nile virus is transmitted between birds by enzootic, ornithophilic mosquito vectors and to humans and horses primarily by bird-to-mammal bridge vectors, such as *Culex *mosquitoes [5,7]. Several environmental factors including landuse, climate, and host community composition can influence the abundance of WNV hosts and vectors, and subsequently, affect WNV transmission rates. For example, increased temperatures from April through October and increased agricultural activities enhanced human WNV disease prevalence in Colorado, Louisiana, Nebraska, and Pennsylvania [1]. Also, increased percentage of urban land or decreased percentage of forest land increases the human WNV incidence in the northeastern United States [8]. Finally, increased avian species diversity was associated with reduced WNV host prevalence and consequently lower WNV transmission rates throughout the United States [9]. However, Yiannakoulia and Svenson [10] found that inclusion of data on corvids did not improve predictive powers of their models for WNV spread in Alberta, Canada.

The role of precipitation in the WNV transmission is inconclusive. It is generally thought that precipitation limits mosquito abundance and that mosquito populations are positively correlated with precipitation. However, Miramontes et al. [1] found no association between annual precipitation and human WNV incidence. Surprisingly, Chase et al. [11] found that mosquito outbreaks were associated with droughts during the previous year, suggesting that droughts reduce mosquito predators and competitors, allowing mosquito abundance to increase the following year. Although it seems counterintuitive, it is plausible to extend this reasoning and hypothesize that dry weather or low precipitation would increase the number of the human WNV incidences the following year due to increased mosquito abundance. It is known that periodic droughts are associated with certain climate regions and with global teleconnection phenomena such as the El Nino-Southern Oscillation; thus, knowledge of relationships between precipitation and WNV transmission is crucial for predicting the risk of the human WNV disease outbreaks under predicted global climate change scenarios. However, the hypothesis regarding the effects of dry weather on WNV transmission has not been tested empirically.

Spatial heterogeneity exists in the risk of human exposure to infectious disease vectors [8,9,12]. For example, occurrences of infectious diseases often are spatially (auto-) correlated; human disease incidence at a location is positively related to incidences at neighboring locations. Therefore, models using geospatial statistics and disease mapping methods are necessary to explicitly estimate the influence of neighboring site's risks on the risk at a specific site [13,14].

We developed a Bayesian hierarchical spatial model with conditional autocorrelation (CAR) distributions to estimate the relative risk of human WNV infection in Mississippi, the United States of America (USA), and account for spatial autocorrelations. Mississippi had the highest human incidence rate (57 cases per million people) in the 2002 WNV epidemic in the US [3]. Our objective was to test the hypothesis that dry weather would induce outbreaks of human WNV the following year, assuming that dry weather-induced increases in mosquito abundance would increase WNV transmission from birds to humans.

## Methods

### Human incidence data and expected number of human West Nile virus cases

We used numbers of human WNV cases (*O*) by county in Mississippi in 2002 http://ems.msdh.state.ms.us/msdhsite/_static/resources/524.pdf. Overall, 193 human cases of WNV were reported in 50 of the 82 counties in Mississippi. The expected counts or rates of human WNV cases (*e*) of a county was estimated with the equation  following the method described by Lawson et al. [14], where *r*_*i *_is the 2002 US national human incidence rate by age group (*i*) [3], and *N*_*i *_is the number of people by age group for the county from the 2000 US Census data obtained from the Mississippi Automated Resource Information System http://www.maris.state.ms.us. This method assumes that age-specific rates of human WNV cases in Mississippi can be approximated by the national age-specific rates. It is possible that some human WNV cases in Mississippi during 2002 were not reported. However, possible additional unreported cases had little effects on our estimates of relative human WNV risk because the same possibility existed in the 2002 national data that were used to compute national human incidence rates.

### Weather data

Seventy three Mississippi counties had at least one weather station. We used the total annual precipitation recorded at a weather station during 2001 to represent the annual precipitation for an entire county. We interpolated annual precipitation for the 9 counties missing precipitation records during 2001, using an ordinary kriging method [15]. Briefly, we first occularly fitted a semivariogram model to the observed data to generate initial values of the parameters range and sill. We then fitted a semivariogram model using the maximum likelihood function and the initial values of range and sill. For kriging, we employed the Marten function as a spatial correlation function and varied values of the order parameter (*κ*) to maximize the model likelihood [15]. The predicted annual precipitations at the centroids of the 9 counties were then used to interpolate missing annual precipitation.

### Bayesian hierarchical models for relative human West Nile virus risk

We assumed that the observed number of human WNV cases had a Poisson distribution. The mean number of human WNV cases reported (i.e., the Poisson parameter) is the product of the expected rate (*e*) and relative risk (*θ*) in a county, i.e. *O*_*i *_~ *pois (λ*_1_) and *λ*_*i *_= *e*_*i*_*θ*_*i*_, where *λ*_*i *_is the Poisson parameter; *e*_*i *_is the expected rate of the ith county, representing the background population effect; and *θ*_*i *_is the relative risk. The expected rate is assumed to be known. The relative risk, *θ*, is either a constant or a function of environmental variables. If the value of *θ *is greater than 1, the risk of disease is greater than expected based on information from the standard population.

To test our hypothesis regarding the effects of previous precipitation on the human WNV epidemic, we developed a set of candidate models with and without precipitation using the logarithmic link function,(1)(2)

where *α *is an intercept representing a fixed, population-level effect; *β *is the regression coefficient; *prec*_*i *_is the annual precipitation during the previous year; *v*_*i *_is a spatially independent random variable normally distributed, *v*_*i *_~ *N *(0, ), representing spatially uncorrelated heterogeneity (UH); and *u*_*i *_is spatially-structured random variable representing effects of (the first-order or directly bordering) neighboring counties on the relative risk. In our preliminary analysis, a model including precipitation of both the previous and the current years did not explain more variability than did a model only including precipitation of the previous year. We aimed to test the predefined hypothesis regarding the time-lag effect of precipitation [11]; thus, we did not include precipitation of the current year in our analysis. We used conditionally autocorrelative distributions (CAR) to model the spatially correlated heterogeneity (CH) in the relative risk [14]. We built our candidate models and estimated the unknown parameters and the UH and CH effects within the framework of Bayesian hierarchical models using the following prior distributions assigned,

*α *~ *flat *( ),

*β *~ *N *(0, ),

 ~ inverse gamma (0.5, 0.0005),

 ~ inverse gamma (0.5, 0.0005).

These priors are relatively non-informative. The hierarchical models were implemented with the program WinBUGS 1.4.3 [16]. A sample WinBUGS code was presented in Appendix A. We initialized two chains for parameters *α *and *β *with different starting values and convergence was assessed by the Brooks-Gelman-Rubin method offered in WinBUGS [16]. The MCMC chains were run for 20,000 iterations with the first 10,000 iterations as a burn-in period. Although we were not able to test the assumption that the observed number of human WNV cases had a Poisson distribution in the Bayesian context, extra variability that often was observed in the number of the infection incidences of an infectious disease was accounted for by the unstructured and structured heterogeneity *v*_*i *_and *u*_*i *_in models (1) and (2). When information about reported infections is known at given time points, a Poisson distribution may be valid for infectious diseases [14]. Poisson distributions were applied to incidence data of infectious diseases in the literature [17,18]. Moreover, we used estimated precipitation by the kriging interpolation for missing data in nine counties. Estimation uncertainty of missing precipitation and its effects on estimates of the parameter *β *measuring rain effects were not assessed in this study.

We created a set of 5 models according to pre-defined hypotheses in a forward stepwise manner. We used deviance information criterion (DIC) to select the best approximating models from the candidate models [19]. The lower the DIC value, the better the model fit. We also used Moran's *I *[18] for the Poisson data to assess goodness of fit. A significant Moran's *I *indicates a lack of fit due to spatial autocorrelation.

## Results

**Figure 1:**
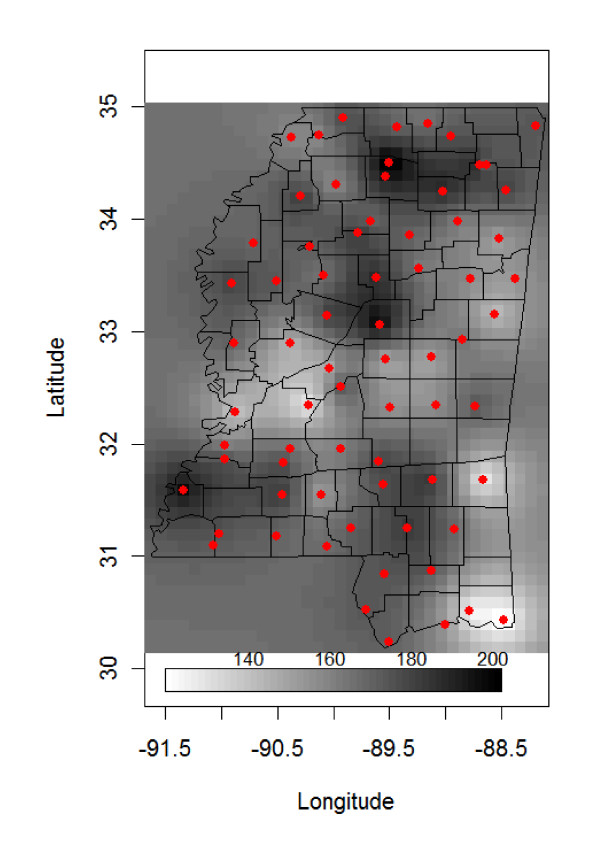
**Kriging of annual precipitation (cm) during 2001 using data from 73 weather stations (circles) in Mississippi, the United States**.

**Figure 2:**
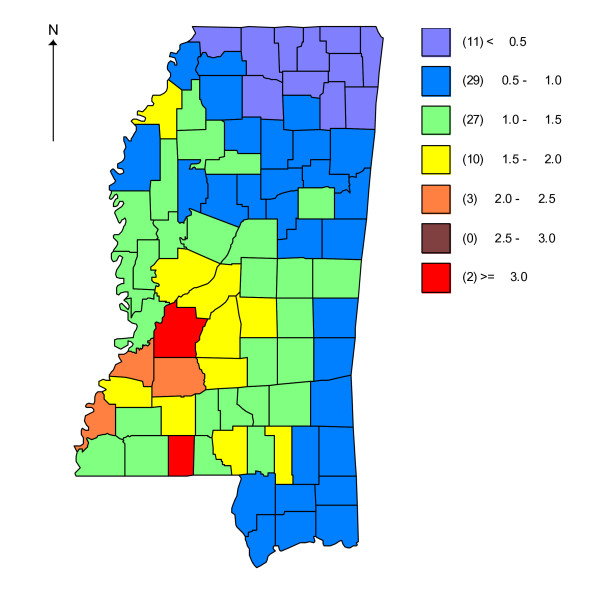
**Relative risk of human West Nile virus of 2002 in Mississippi, the United States, estimated by Bayesian hierarchical models**. Counties with values exceeding 1 have greater than expected risk.

Annual precipitation varied across Mississippi in 2001, ranging from 111.9 cm to 208.1 cm (Figure 1). Central and western Mississippi received less precipitation than northern Mississippi. Smoothed estimates of the relative risks of human WNV from Eqn 1 also suggested considerable spatial variation across Mississippi with the greatest human WNV risk in west central Mississippi. The areas of highest relative risk ( >1.0) during the 2002 human WNV epidemic generally received less precipitation during 2001 (Figure 1, 2).

Annual precipitation during the previous year improved model fit and reduced the DIC value by 17.19 compared to the Poisson constant model (Table 1). The 95% credible interval (CI) of coefficient *β *ranged from -0.02 to -0.007, indicating an inverse relationship between the relative risk of human WNV and annual precipitation during the previous year. However, the Poisson regression model without UH and CH components was insufficient; Moran's *I *had a 95% CI from 0.07 to 0.1. The UH and CH components substantially improved model fitting and resulted in lower DIC values (Table 1). Models 1 and 2 were competing models with ΔDIC <0.6. Although the 95% CI included zero, most of the CI range was negative, suggesting an inverse relationship between the relative risk of human WNV and annual precipitation during the previous year.

**Table 1:** Bayesian hierarchical models for the effects of annual precipitation on the relative risk of human West Nile virus in Mississippi, the United States.

Model	DIC	ΔDIC	Weight	Mean and 95% CI of precip coefficient
*c*	326.77	76.73	0.00	NA
*c *+ Precip	309.58	60.54	0.00	-0.014 (-0.020, -0.007)
*c *+ Precip + UH	257.80	8.76	0.01	-0.008 (-0.020, 0.005)
*c *+ Precip + UH + CH	249.58	0.54	0.43	-0.005 (-0.020, 0.008)
*c *+ UH +CH	249.04	0.00	0.56	NA

Note: letter *c *denotes constant; Precip is annual precipitation of the previous year; UH is uncorrelated heterogeneity; CH is the correlated heterogeneity modeled by conditional autocorrelative distribution; DIC is deviance information criterion; ΔDIC is the difference between the DIC of a candidate model and the lowest DIC; weight is equivalent to Akaike weight; and CI is credible interval.

## Discussion

West Nile virus is amplified in its avian hosts and is transmitted to humans by *Culex *mosquitoes. Abundance of mosquito larvae is inversely related to the presence and abundance of predatory fish [11]. Droughts presumably reduce predators and competitors of mosquitoes, allowing mosquito abundance to increase [11]. In a survey following a drought, Chase et al. [11] found a significant decline in the biomass of mosquito's predators and competitors in dried natural wetlands compared to that in permanent wetlands. Drought-induced declines in the predator and competitor biomass of mosquitoes and subsequent increase in mosquito abundance were confirmed in a controlled mesocosm experiment [11]. In addition, *Culex *mosquitoes are a "foul water species," thriving in dry conditions by breeding in standing water in old tires or pooled in tire tracks [10,20]. Future studies should address the effects of dry weather conditions on the abundance of the predators and competitors of mosquitoes to better understand climate effects on human WNV risks.

Increased bird abundance at refuges with congregations of *Culex *mosquitoes during droughts was suggested to amplify the transmission risk of St. Louis Encephalitis virus (SLEV) [21]. Dispersing mosquitoes and SLEV-infected birds were associated with increased risk of SLEV infection in humans [21]. The inverse relationship between annual precipitation of the previous year and the relative risk of human WNV suggests that drought-induced increases in mosquito abundance ultimately increase the risk of transmitting WNV from birds to humans. Therefore, drought-induced mosquito outbreaks and congregations of WNV-infected mosquitoes and birds are potential mechanisms for increased post-drought WNV risks. Post-drought recolonization and dispersal of infected mosquitoes and birds may in part have caused the observed time lags between droughts and WNV epidemics.

Landuse can alter the suitability of mosquito and bird habitats and consequently influence the transmission of WNV among birds and to humans [8]. Deforestation increases water surface runoff and water table levels which in turn can improve habitat suitability and increase mosquito densities [22,23]. Consequently, urbanization and reduction of forest cover can increase the risk of human WNV incidence [8]. The central part of Mississippi, including the Jackson area, is the most densely populated portion of the state and had a high relative risk of human WNV. Our models included the expected rate of human WNV, which was a function of the population size of a county and represented the expected number of people exposed to WNV. However, it is unknown whether the higher risk around the Jackson area of Mississippi was due to urban development or the population disparity with surrounding areas. Yiannakoulias et al. [10] found that estimating the spread of WNV without including landuse (i.e., urban, rural, and natural areas) was potentially biased. Furthermore, reductions in water table levels may fragment mosquito habitat with remaining fragments providing refuges for mosquitoes and birds to congregate. Loss of bird habitat due to forest fragmentation can increase avian densities within remaining fragments and may facilitate the transmission and amplification of WNV. Therefore, assessments of the effects of bird habitat fragmentation on WNV risk and other avian transmittable diseases are warranted.

Moran's *I *of the Poisson constant model (the first model of Table 1) was significant; thus, the CAR distribution was needed to model the correlated structure in human WNV counts. However, a mixed model of the CAR distribution often results in poor estimation of covariate effects in a linear relationship even when the relationship is actually strong [24]. Although the 95% CI of coefficient *β *in the competing models 1 (Precip + UH) and 2 (Precip + UH + CH) included zero, most of the 95% CI was negative (Table 1). Additionally, the DIC difference between the best model without precipitation and the second best model including precipitation of the previous year was very small (0.54). We concluded that the model (Precip + UH + CH) received considerable support by data with the deviance weight of 0.43. Derived from SMR models and the models (1) and (2), relative human WNV risk (*RR*) of a county was predicted by the nonlinear equation , where *preci*_*i *_is the annual precipitation deviation from the mean annual precipitation over all the Mississippi counties. An increase of 10 cm in annual precipitation from 120 cm resulted in a 10% decrease in predicted relative risks. During 2001, annual precipitation changed substantially from county to county in Mississippi, from the minimum of 120.8 cm in Jackson County to the maximum of 208.9 cm in Lafayette County. The relative risk model predicted a 143% decrease in human WNV relative risk when annual precipitation of a county changed from the maximum to the minimum. Therefore, annual precipitation of previous year was a significant predictor of human WNV risk. The relatively poor estimation of the effects of precipitation on the relative risk of human WNV in the UH and CH model might be in part due to poor representation of annual precipitation within a county. We used precipitation data from one weather station for each county, which might be insufficient to estimate annual precipitation for the entire county. Furthermore, precipitation may interact with landscape variables to affect the transmission of WNV.

## Conclusions

Our results have broad implications for the assessment of WNV risk and forecasting WNV outbreaks. Delayed effects of dry weather on the WNV epidemic provided an opportunity to forecast the human WNV outbreak in Mississippi USA. As the magnitude and frequency of droughts are predicted to increase from global warming, our work and previous studies [11,21] suggest the risk of human WNV will also increase. Moreover, the counter-intuitive effects of dry weather on WNV risk reiterate the importance of interspecific interactions or food-web theory in the studies of climate effects on vector-born infectious disease [6,11,25].

## Competing interests

The authors declare that they have no competing interests.

## Authors' contributions

GMW and RBM designed the study. GMW carried out the statistical analysis. GMW, RMM, JLB, and CLW wrote the manuscript. All authors read and approved the final manuscript.

## Appendix

A. Sample WinBUGS code for the estimation of relative human West Nile virus risk using conditional autoregressive distributions for spatial autocorrelation and annual precipitation as an independent variable.

model {

   d<-mean(X[]) #X[] is a vector of annual precipitation

   for (i in 1 : N) { #N is the totoal number of counties

      O [i] ~ dpois(mu[i]) #O[] is a vector of observed numbers of human WNV incidences

      log(mu[i]) <- log(E[i]) + alpha0 +c*(X[i]-d)+b[i]+u[i]

      RR[i] <- exp(alpha0 +c*(X[i]-d)+ b[i]+u[i]) # RR[] is county-specific relative risk

      u[i]~dnorm(0, tau1)

   }

   # CAR prior distribution for correlated heterogeneity

   b[1:N] ~ car.normal(adj[], weights[], num[], tau)

   for(k in 1:sumNumNeigh){

      weights [k] <- 1

   }

   # Other priors:

   alpha0 ~ dflat()

   c~dnorm(0,0.000001)

   tau1 ~ dgamma(0.5, 0.0005)

   tau ~ dgamma(0.5, 0.0005)

}

## Pre-publication history

The pre-publication history for this paper can be accessed here:

http://www.biomedcentral.com/1471-2334/10/38/prepub
